# Perception and Awareness about Monkeypox and Vaccination Acceptance in an At-Risk Population in Brescia, Italy: An Investigative Survey

**DOI:** 10.1007/s10461-024-04271-9

**Published:** 2024-01-19

**Authors:** Verena Crosato, Beatrice Formenti, Maurizio Gulletta, Silvia Odolini, Silvia Compostella, Lina Rachele Tomasoni, Alberto Matteelli, Francesco Castelli

**Affiliations:** 1 Division of Infectious and Tropical Diseases, University of Brescia and ASST Spedali Civili Hospital, Brescia, Italy; 2 UNESCO Chair in Training and empowering human resources for health development in resource-limited countries, University of Brescia, Brescia, Italy; 3 Division of Clinical Psicology, ASST Spedali Civili Hospital, Brescia, Italy; 4 Division of Infectious and Tropical Diseases, Department of Clinical and Experimental Sciences, University of Brescia, Brescia, Italy

**Keywords:** Monkeypox, Awareness, Perception, Risk population, Survey, Outbreak

## Abstract

Before 2022, monkeypox virus (Mpox) infection in humans was seldom reported outside Africa. During the May 2022 outbreak, most cases were detected among men who have sex with men (MSM). Since Mpox is largely unknown to the general population, through a self-completion questionnaire, we investigated the behaviours and knowledge of our at-risk population belonging to the sexually transmitted infection (STI) outpatient clinic of the Infectious Diseases Unit of the ASST Spedali Civili of Brescia, Italy, between August and October 2022. Most patients that took part in the compilation are HIV positive MSM. The other participants were HIV-seronegative patients with other STIs. Overall, 144 questionnaires were compiled. Most of the participants were Italians (130;90%) and males (139;96.5%) between 30 and 60 years (118;82%). Almost all (136;94%) reported having heard about Mpox and more than half (80;56%) received information about the transmission. Twenty-four respondents (16%) received information from health professionals and 14 (10%) believed that the information received was complete. Although 41% of respondents thought they were at risk of getting the infection and 62% were afraid to get it, the majority (56%) did not increase the precautions taken. When asked if they would accept a vaccine to prevent the disease, more than a third (32%) of respondents expressed hesitation or complete refusal to be vaccinated. Based on our results, what emerges is that there is still a lack of knowledge and awareness about Mpox. To address this issue, targeted health promotion and education strategies that provide the necessary resources to reduce risk behaviours and enhance connections with healthcare professionals are needed.

## Introduction

Before 2022, monkeypox virus (Mpox) infection in humans was primarily found in Central and West African regions where the virus is endemic. Human-to-human transmission of Mpox is commonly attributed to respiratory droplets or direct contact with mucocutaneous lesions of an infected individual [1]. Despite the virus being present in endemic regions for many years, research into Mpox has been largely neglected.

However, since May 2022, the World Health Organization declared it an “evolving threat of moderate public health concern” after over 3,000 cases were reported in more than 50 countries across five regions [2]. 

Since the onset of the Mpox outbreak, and as of 29 March 2023, a total of 86.746 cases have been identified across 45 countries and regions within the European Region [3]. The majority of cases were reported among individuals between the ages of 31 and 40, with a higher prevalence among males. Notably, men who have sex with men (MSM) have been disproportionately affected, suggesting an amplification of transmission through sexual routes. Moreover, among cases with available HIV status information, 38% of the infected individuals were found to be HIV-positive [3]. 

The increase in the number of monkeypox cases has coincided with a significant surge in the dissemination of misinformation through social media platforms, which are increasingly utilised as health research tools [4]. This misinformation has led to stigmatisation and undermined adherence to public health guidance. To combat the deleterious effects of this “misinfodemic,” it is essential to gauge the population’s perceptions and level of awareness of monkeypox. Stigma during the Mpox pandemic drew parallels with the long-standing discrimination and mistreatment that people with HIV have faced for years. Both epidemics show “othering,” creating stigma that leads to social isolation and mistreatment, limiting access to resources and opportunities, social cohesion, and hinder disease prevention efforts [5]. Stigma can also have negative effects on mental health and may lead to a decline in testing during outbreaks. In the case of Mpox, stigma could discourage individuals from seeking help or getting vaccinated [6]. 

Limited research has concentrated on understanding the awareness and perceptions of Mpox within the population at higher risk of contracting it. Several surveys have explored Mpox knowledge among healthcare professionals and university students in non-endemic countries. Even in this group, findings indicate a suboptimal level of knowledge [7, 8]. 

To date, very few studies have investigated the level of awareness and actions taken by the population at risk of contracting the disease and the connection between their behaviour and other important factors, such as level of education and main source of information. Notably, these two factors proved pivotal during the recent SARS-CoV-2 pandemic, especially in terms of acceptance of the regulations and vaccinations.

Identifying and evaluating factors the general knowledge and awareness of Mpox in at-risk population groups is crucial in developing targeted and effective strategies to address this infection.

## Objective

We report here the results of an investigation to elucidate perception and knowledge on Mpox in an at-risk-population. In addition, we assessed how having received information from healthcare personnel may influence the knowledge of Mpox and willingness to undergo vaccination.

## Materials and Methods

This is a cross-sectional observational study conducted between August and October 2022 and based on a self-administered anonymous questionnaire drafted by the Department of Infectious and Tropical Diseases of the ASST Spedali Civili of Brescia, Italy.

### Population

Our study population is constituted by persons presenting to the sexually transmitted infection (STI) outpatient clinic of the Infectious Diseases Unit of the ASST Spedali Civili in Brescia. The patient cohort attending this clinic is predominantly composed of PLWH or HIV-seronegative patients with STIs, such as syphilis, gonorrhoea and chlamydia, therefore considered an at-risk population group for contracting Mpox.

### Data Collection

A paper-based questionnaire was prepared by physicians of the unit of Infectious Diseases with the support of a clinical psychologist. The questionnaire was distributed to patients prior to their scheduled medical consultation. The self-administered anonymous questionnaire consisted of 29 multiple choice questions, which covered the following main topics: demographic information (Gender, Age, Nationality, Education, Level, Occupation); awareness and knowledge (16), fear and stigma (6) and vaccination acceptance (1).

### Analysis

We performed a descriptive analysis describing frequencies of categorical variables. Fisher’s exact test was used to compare outcomes (awareness of Mpox as a dangerous disease, knowledge of preventive measures and willingness to undergo vaccination) between participants who have received specific information from health personnel; differences were considered significant when *p* < 0.05 (Epi Info 7.2 software).

## Results

### Characteristics of the Population

All individuals who were invited to participate in the study accepted and completed the questionnaire, with a response rate of 100%. Overall, 144 questionnaires were collected. The vast majority of the participants were of Italian nationality, (130;90%), of male gender (139;96.5%), and between 30 and 60 years of age (118;82%). Foreigners were mainly from South America (10;71.4%). For the majority (65;45.1%), the highest educational attainment was a high school diploma, while 34 (23.6%) participants held a bachelor’s degree. The vast majority were employed (121;84%), with the remaining individuals being either unemployed, retired, or still students.

### Perception and Awareness

Almost all participants (136;94%) reported having heard about Mpox before, and more than half (80;56%) had received information about transmission. The information was perceived as complete by 14 respondents (10%). The main source of information was television and newspapers (118;82%), followed by the websites and social media (71;49%), healthcare professionals (24;16%) and finally word-of-mouth (14;10%).

The vast majority of participants (109;76%) believed that Mpox is a dangerous disease, mainly because it is an infectious disease (93;65%) and because it causes visible symptoms (28;19%), while few thought that it is incurable (7;5%). In fact, the majority (93;65%) think that there is a specific drug to cure Mpox. A third (45;31%) thought it is easy to acquire the infection, while half (76;53%) were unaware.

The majority of the cohort knew that transmission occurs through direct contact with an affected person (100;69%), and half also stated through sexual route (75;52%). The transmission route via respiratory droplets (20;14%), kissing (20;14%) and through objects (19;13%) and contaminated surfaces (2;1%) was the least selected.

Nearly half (59;41%) of respondents affirmed they thought they were at risk of getting the infection, and the majority (89;62%) were afraid to get it, mainly out of fear of a disease that is still little known, and out of social stigma and fear that the signs of infection may be visible.

The majority of participants (91; 63.2%) believe that friends and family members would react with negative emotions if they were to discover that they suffer from Mpox.

Despite the fear of the infection, the majority of participants (81;56%) responded that they did not increase the precautions they took for fear of Mpox. Indeed, most respondents (86;59.7%) declared they did not know what preventive measures to take to reduce the risk of infection.

Furthermore, less than half (61;42%) answered that using condoms could reduce the risk of infection, and the great majority (123;85%) do not think that hand washing is a proper Mpox prevention rule.

When asked if they would accept a vaccine to prevent the disease, more than a third (46;32%) of respondents expressed hesitation or complete refusal to be vaccinated. All these results are summarized in Table 1.

**Table 1:** Responses of participants to the questionnaire. Percentages are calculated on total participants

Demographics	N (%)
Sex	
Male	139 (96.5%)
Female	4 (2.8%)
Other	1 (0.7%)
Age	
30–60 years of age	118 (81.9%)
> 60 years of age	17 (11.8%)
Nationality	
Italian	130 (90.3%)
Foreign born	14 (9.7%)
Of which, from South America	10 (71.4%)
Education	
Secondary school	41 (28.5%)
High school	65 (45.1%)
Degree	34 (23.6%)
Occupation	
Employed	121 (84%)
Unemployed	9 (6.3%)
Retired	11 (7.7%)
Student	3 (2%)
**Monkeypox perception and knowledge**	
Have you ever heard about monkeypox?	
Yes	136 (94.4%)
No	8 (5.6%)
Have you ever received information about the transmission?	
Yes	80 (55.6%)
No	63 (43.8%)
What was the source? (Multiple answer)	
Health-care personnel/setting	24 (16%)
Web and social media	71 (49.3%)
Television and Newspapers	118 (81.9%)
Word-of-mouth	14 (9.7%)
The information received were (Multiple answer)	
Completed	14 (9.7%)
Sufficient	58 (40.3%)
Insufficient	43 (29.9%)
Comprehensible	21 (14.6%)
Incomprehensible	11 (7.7%)
Do you think monkeypox is a dangerous disease?	
Yes	109 (75.7%)
No	32 (22.2%)
Why do you thing is a dangerous disease? (Multiple answer)	
It is an infectious disease	93 (64.6%)
It is incurable	7 (4.9%)
It is sexually transmitted	29 (20.1%)
It causes visible symptoms	28 (19.4%)
Do you think it is pharmaceutically treatable?	
Yes	93 (64.6%)
No	6 (4.1%)
I don’t know	44 (30.6%)
In your opinion, is it easy to catch monkeypox?	
Yes	45 (31.3%)
No	22 (15.3%)
I don’t know	76 (52.7%)
How is monkeypox transmitted? (Multiple answer)	
By contact with infected persons	100 (69.4%)
By respiratory droplets	20 (13.8%)
By using contaminated items	19 (13.2%)
By contaminated surfaces	2 (1.4%)
By kissing	20 (13.8%)
By sex	75 (52%)
Do you know what preventive measures should be taken?	
Yes	55 (38.2%)
No	86 (59.7%)
How do you think it is possible to reduce the risk of monkeypox transmission? (Multiple answer)	
In no way	7 (4.9%)
Always using a condom	61 (42.4%)
Frequently washing hands	21 (14.6%)
Wearing a mask in public places	16 (11.1%)
Having only one sexual partner	38 (26.4%)
Avoiding casual sexual encounters	47 (32.6%)
Not exchanging personal items	20 (13.9%)
Do you usually take precautions against STIs?	
Habitually	112 (77.8%)
Sometimes/rarely	20 (13.9%)
No, never	4 (2.8%)
Since the emergence of monkeypox, are you taking more precautions?	
Yes	54 (37.5%)
No	81 (56.3%)
Are you afraid of being affected?	
Yes	89 (61.8%)
No	30 (20.8%)
I don’t know	18 (12.5%)
Have you ever thought you were at risk of getting monkeypox?	
Yes	59 (41%)
No	76 (52.8%)
How do you think family and friends would react if they knew you were affected by monkeypox? (Multiple answer)	
Negatively (they would isolate me, judge me)	91 (63.2%)
They would support and help me	31 (21.5%)
No reaction	35 (24.1%)
If a vaccine was available, would you get it?	
As soon as possible	86 (59.7%)
I would wait some time to see the effect on the people who did it	40 (27.8%)
I would never do that	6 (4.2%)

Significant differences were found in responses between participants who received medical information from health professionals and those who did not. The vast majority of those who received information from health professionals knew the preventive measures to be taken to avoid infection (15;62% vs. 40;36%, *p* = 0.022). Moreover, the first group declared that since the emergence of Mpox virus they had been taking more precautions against sexually transmitted infections (14;63% vs. 38; 35%, *p* = 0.017). However, we didn’t find significant differences on the acceptance or refusal of vaccinations between patients who received information about Mpox from healthcare professionals and those who did not (16;72% vs. 70; 66%, *p* = 062), as well the awareness of the danger of the disease (19;82% vs. 85;75%, *p* = 0.593). These results are represented in Table 2.

**Table 2:** Differences between answers in participants who acquired information from healthcare professionals and participants that got information elsewhere. Significance is established at *p* < 0.05

Monkeypox perception and knowledge	N (%)	*p*-value
Monkeypox is a dangerous disease		
Healthcare personnel as source of information	19 (82%)	*p* = 0.593
Others	85 (75%)	
Knowledge of the preventive measures		
Healthcare personnel as source of information	15 (62%)	*P* = 0.022*
Others	40 (36%)	
Taking more precautions against IST since the emergence of Mpox virus		
Healthcare personnel as source of information	14 (63%)	*P* = 0.017*
Others	38 (35%)	
If the smallpox vaccine becomes available, I would like to receive it as soon as possible		
Healthcare personnel as source of information	16 (72%)	*P* = 0.624
Others	70 (66%)	

## Discussion

The results of this study highlight a concerning lack of knowledge and awareness about Monkeypox among a cohort of individuals followed by an STI clinic, mostly composed of HIV-positive MSM, who are considered a population at risk for acquiring the infection. According to available literature, despite the use of multiple scales to assess the level of knowledge about Mpox in various studies, the results have generally indicated a poor or moderate level of knowledge [9], even among healthcare professionals and university students [7]. However, surveys on knowledge and awareness on Mpox targeting “at risk” communities are still scarce.

Despite the perceived severity of Mpox, a significant proportion of respondents did not increase the precautions they took to avoid infection and were unsure of the preventive measures to be taken. This lack of awareness and knowledge may contribute to a higher risk of infection, particularly given that more than a third of respondents expressed hesitation or refusal to accept a vaccine to prevent the disease. This data is concerning as it highlights that a significant portion of the population still requires awareness on the importance of vaccination. This data is in line with a survey in which only nearly half of MSM living with HIV in China were willing to get the Mpox vaccine [10]. The fact that no significant differences were found between patients who received information about Mpox from healthcare professionals and those who did not suggests that current efforts to disseminate information and promote vaccination may not be sufficient and that additional strategies are needed to increase vaccine uptake and improve knowledge about Mpox. A systematic review and meta-analysis have revealed that the general population had a low acceptance rate of Mpox vaccines (43%), whereas the acceptance rate was higher among the lesbian, gay, bisexual, and transgender (LGBTI) population (84%). As such, efforts should be made to prioritise education and promotion about Mpox, including transmission and preventive practices, within LGBTI communities, while stigma should be avoided [11].

While the majority of participants to our survey reported having heard about Mpox before, only a small percentage claimed to have received information about Mpox from healthcare professionals, and many did not believe the information they received was complete. Most of our respondents declared having received most information from traditional and modern social media. Two studies conducted in 2022, highlight the fundamental role that social media have gained in the last years in the spread of medical information, but also misinformation. In particular, he emphasised the tendency of the general population to retrieve information on Mpox on websites such as YouTube and Tik Tok, and almost never from channels led by healthcare professionals [12, 13]. This trend favours the spread of misleading and sometimes conspiratory theories, at the expense of attempts to improve communication between the scientific community and patients.

In fact, our study found that respondents who had received information from healthcare professionals had better knowledge about transmission and preventive measures and were more likely to take more precautions against sexually transmitted infections since the emergence of Mpox virus. This highlights the importance of healthcare professionals in providing accurate and complete information about Mpox and the need for increased tailored awareness-raising campaigns to target at-risk populations. Admitting the importance of social media in gaining attention from the general public could also be seen as an opportunity by the medical community to gain the trust of the population and spread useful information on current and future topics.

## Conclusions

In conclusion, this study emphasises the critical importance of improving education and awareness strategies related to Mpox among high-risk populations. To achieve this goal, public health campaigns and healthcare providers must play a crucial role in disseminating accurate information and advocating for preventative measures aimed at mitigating the transmission risk of Mpox and other potential public health threats. To this end, the implementation of targeted health promotion and education strategies that facilitate resources for reducing high-risk behaviours and enhancing connections with healthcare professionals is of paramount importance. Such strategies should aim to empower individuals with the knowledge and resources necessary to make informed decisions about their health and wellbeing.
